# Podophyllin

**Published:** 1863-01

**Authors:** J. B. Hoag

**Affiliations:** Nevada, Ind.


### PODOPHYLLIN.

By J. B. HOAG, M. D., Nevada, Ind.

One of the most complete and contemptible farces that has ever been imposed on a credulous community, and one which has already done and is likely to do no little mischief, is that which takes the conspicuous cognomen of "The Reformed

Practice of Medicine." In the first place it had its origin with a complete ignoramus who took the position that all diseases had one origin and one method of cure, and with six articles and combinations, proposed, by the addition of steaming, etc., to be able, by a certain "course of medicine," to cure all diseases. Taking for his basis of reasoning the idea that "heat is life and cold is death," he formed what was termed the "Thompsonian system of medical practice." As absurd and ridiculous as was his theory, and as injurious as the practice necessarily was, it still had its advocates. The writer has a distinct recollection of being once called upon by one of the adherents of this system to administer to the case of his son who was attacked with bilious remitting fever in its worst form. He remarked that no man could practice in his family who bled, blistered or gave calomel. We replied that we could not tell what was indicated until we saw the case. On arriving at the house of our patient we found a high fever and no little delirium. After a careful examination of the case, we remarked: "You can do as you please; employ me or some one else; but if I treat that case I shall use calomel." After pausing a while he replied: "Well, do the best you can." The mother interposed with the objection that she feared that if he took calomel he would be ruined for life. We assured her that she need fear no such result if our directions were followed. We treated the case, and on each subsequent visit found the officious disciple of Thompson was determined to have "a finger in the pie." Not satisfied with having the child literally burning with fever, he had to add a process of cooking in the shape of hot bricks covered with wet cloths placed around the body of the patient. He also urged me to give him, when his fever was at the highest pitch, the strongest preparations of capsicum which I had. I remarked that the arterial circulation was too much excited already and that to give arterial stimulants would probably prove injurious. "Ah, faugh Doctor," he replied, "heat is life and cold is death; raise the heat and there is no danger of dying." "Well," said I, a little provoked, "I know of no better way to carry out your theory than to place him on the back log of the fire, and unless you cease interfering I will cease to treat the case." He ceased to meddle, except occasional steaming, and the boy recovered, and then he boasted that "*we* cured him;" but I always believed if nature had sunk under his barbarous interference it would have been that calomel doctor killed him.

This system was succeeded by the Eclectic system, but little less absurd, which consists mainly in declaiming against mercurial preparations and mineral poisons, while their substitutes were not less objectionable. It has opened a wide door for quackery, and imposed a set of unqualified pretenders on the community, men destitute of scientific knowledge of medical skill. We conceive, however, that with all the harm that has been done by seeking to overthrow the splendid structure of medical science which has been reared by ages of devotion by the most talented and learned men the world ever saw, and which will ever stand as a lasting monument to their talents and worth, one essential good has been achieved. In their desire to present to the world something new, to be able to boast of something original, the advocates of the so-called reform school of medicine have necessarily been driven to investigations and experiments which have resulted in many valuable additions to the Materia Medica. By this means the medical properties of many vegetables which were heretofore unknown have been discovered and their usefulness tested, and many beneficial medical agents placed in the hands of the medical profession. We apprehend that the work of making additions to the Materia Medica from the vegetable kingdom is far from being completed yet, but that the time is not far distant when many of the plants that strew our earth, which are now disregarded, will take their places with the list of remedial agents and be numbered among the most valuable which the medical profession are in possession of.

Previous to the rise of the Eclectics the Podophylum Peltatum was rarely used as a medicine. The quantity required for a dose, in order to have any effect, was an objection to its use; but since chemists have extracted the *resinous* principle from the plant it is much more generally used.

The pretentions of the Eclectic fraternity, that it is a substitute for and capable of taking the place of mercurial preparations, is all folly. Yet it has its uses, and if properly prepared and correctly used may prove a valuable remedial agent in the hands of the skillful practitioner. For the last seven years we have made a somewhat extensive use of it and have tested its nature thoroughly, and hesitate not to say that in many complaints it is truly valuable. It seems to be peculiarly adapted to cleansing the intestinal tube of vitiated matter, with which it frequently becomes overcharged and loaded, especially in localities which are subject to bilious diseases, and all diseases which accompany torpidity of the liver. The fact that it sometimes produces nausea and even vomiting, is urged by some as an objection; but we have found this to be the case only when the "stomach was foul" and the system generally deranged by the presence of a large amount of vitiated matter.

We certainly know of no medicine which will so effectually clean and clear out the mucous membrane of the intestinal tube, as proper doses of Podophyllin properly combined with other medicines. In order to arouse the liver and at the same time clear out the system, we have found it beneficial to combine it with Blue Mass. The following is an excellent formula: ℞—Blue Mass ʒjj; Podophyllin ʒjj; Carb. Soda ʒj; Pulv. Capsicum grs. x vel Fluid Ext. Capsicum gtts. v. M—Ft pill No. 40. One or two to be given every two hours until it operates, and it is well to continue as long as the passages present a bad appearance, if the strength of the system will permit. The capsicum prevents its having any unpleasant effect by griping.

Another excellent formula for the same purpose is the following: ℞—Calomel ℈j; Podophyllin grs. jjj; Leptandrin grs. x; Carb. Soda ℈j; Capsicum grs. v. M—Ft chart No. 3. One to be given every four hours till it operates sufficiently. Or this: ℞—Hydrarg Cum Creta ℈jj; Carb. Soda ℈j; Podophyllin grs. v; Leptandrin grs. xjj; Capsicum grs. v. M—Ft chart No. 4. Given as above.

I cannot but recommend it to the favorable consideration of those practitioners who have never tried it.

### OLD THINGS BECOME NEW.

Messrs. Editors:—In a recent No. of the London *Lancet*, under the head of *New* Remedies, you will find a notice of Gelseminum Sempervirens or Yellow Jasmine, with the following *very flattering compliment to American Physicians:* "Amongst these (that is new American remedies), there are several deserving the attention and careful experiment of English practitioners. As we have remarked already, we *cannot attach the same importance to the testimony of American physicians*, to the remedial properties of these drugs as we do to that offered by practitioners at home." Passing by the story of the Mississippi planter and the wonders of Eclecticism, we may refer the editors of said *Lancet* to the writings of one Charles Linnæus, of Sweden, who published a systematic treatise on *Materia Medica* in 1749, and two years later, his *Philosophia Botanica*, wherein is described the veritable Galseminum (at that time) Officinale, or Yellow Jasmine, with its properties. Before the close of the eighteenth century it was nearly abandoned. Motherby says of it: "it is sufficiently known not to need a description, the flowers now only are in use, and they to perfume insipid expressed oils.

### VERBASCUM THAPSUS.

This will be found well described by one Pedacius Dioscorides, in his *Flora Græcæ Prodr.*, Lib. IV, Cap. 104, as *Phlomos leuki e arren*, who is believed to have written and practised in the reign of one Nero, and for sixteen hundred years was regarded as the first authority in *Materia Medica*. The properties claimed for it, as new, seem to have been known about nineteen hundred years. P. J. Berjius speaks of its narcotic property in his *Materia Medica e Regno Vegetabile*, published in Stockholm, 1778, and those desiring to, will find it prescribed in the writings of the ancients in pulmonary affections, as a narcotic and demulcent, with an ever varying reputation, also used as an emollient.

Are not our astute transatlantic brethren of the London *Lancet* making wonderful progress in introducing new remedies.

JONATHAN W. BROOKS.

Chicago, Illinois, Jan. 16, 1863.

